# The PanCam Instrument for the ExoMars Rover

**DOI:** 10.1089/ast.2016.1548

**Published:** 2017-07-01

**Authors:** A.J. Coates, R. Jaumann, A.D. Griffiths, C.E. Leff, N. Schmitz, J.-L. Josset, G. Paar, M. Gunn, E. Hauber, C.R. Cousins, R.E. Cross, P. Grindrod, J.C. Bridges, M. Balme, S. Gupta, I.A. Crawford, P. Irwin, R. Stabbins, D. Tirsch, J.L. Vago, T. Theodorou, M. Caballo-Perucha, G.R. Osinski

**Affiliations:** ^1^ Mullard Space Science Laboratory, University College London, Dorking, UK.; ^2^ Centre for Planetary Science at UCL/Birkbeck, London, UK.; ^3^ Institute of Planetary Research, German Aerospace Centre (DLR), Berlin, Germany.; ^4^ Space Exploration Institute, Neuchâtel, Switzerland.; ^5^ Joanneum Research, Graz, Austria.; ^6^ Department of Physics, Aberystwyth University, Aberystwyth, UK.; ^7^ Department of Earth and Environmental Sciences, University of St Andrews, St Andrews, UK.; ^8^ Department of Earth and Planetary Sciences, Birkbeck, University of London, London, UK.; ^9^ Space Research Centre, University of Leicester, Leicester, UK.; ^10^ Department of Earth Sciences, Open University, Milton Keynes, UK.; ^11^ Department of Earth Science and Engineering, Imperial College London, London, UK.; ^12^ Department of Physics, University of Oxford, Oxford, UK.; ^13^ European Space Agency, Noordwijk, the Netherlands.; ^14^ Centre for Planetary Science and Exploration, University of Western Ontario, London, Canada.

## Abstract

The scientific objectives of the ExoMars rover are designed to answer several key questions in the search for life on Mars. In particular, the unique subsurface drill will address some of these, such as the possible existence and stability of subsurface organics. PanCam will establish the surface geological and morphological context for the mission, working in collaboration with other context instruments. Here, we describe the PanCam scientific objectives in geology, atmospheric science, and 3-D vision. We discuss the design of PanCam, which includes a stereo pair of Wide Angle Cameras (WACs), each of which has an 11-position filter wheel and a High Resolution Camera (HRC) for high-resolution investigations of rock texture at a distance. The cameras and electronics are housed in an optical bench that provides the mechanical interface to the rover mast and a planetary protection barrier. The electronic interface is via the PanCam Interface Unit (PIU), and power conditioning is via a DC-DC converter. PanCam also includes a calibration target mounted on the rover deck for radiometric calibration, fiducial markers for geometric calibration, and a rover inspection mirror. Key Words: Mars—ExoMars—Instrumentation—Geology—Atmosphere—Exobiology—Context. Astrobiology 17, 511–541.

## 1. Introduction

PanCam is one of the context instruments on the ExoMars rover (Vago *et al.,* 2017, in this issue) and will provide images in the visible and near infrared (NIR), from which crucial observations of landscape morphology, geology, and atmospheric science may be derived. PanCam will be the prime tool to characterize the morphology and geology of rock outcrops on the martian surface and will be crucial during mission operations for geological target selection and characterization. PanCam data will support the overarching exobiological objective of ExoMars through the characterization of paleoenvironments that involved sustained liquid water at the ExoMars study site. The PanCam spectral range is extended by the Infrared Spectrometer for ExoMars (ISEM) instrument (Korablev *et al.,* 2017, in this issue), which will add to the mineralogical determination; as shown by Harris *et al.* (2015), definitive identification of unique minerals is rarely achievable with the PanCam wavelength range alone. In addition, the Close-UP Imager (CLUPI; Josset *et al.,* 2017, in this issue) will provide high-resolution images of potential drilling sites and other interesting samples. Subsurface context will be provided by a ground-penetrating radar (Water Ice and Subsurface Deposit Observation On Mars, WISDOM; Ciarletti *et al.,* 2017, in this issue) and a neutron detector (Autonomous Detector of Radiation Of Neutrons, ADRON; Mitrofanov *et al.* 2017, in this issue), as well as a visible-IR spectrometer (Mars Multispectral Imager for Subsurface Studies, Ma_MISS; De Sanctis *et al.,* 2017, in this issue) in the drill tip to provide *in situ* mineralogy for the subsurface samples before they are brought to the surface. Sample analysis will be by the Analytical Laboratory Drawer (ALD) instruments, MicrOmega (Bibring *et al.,* 2017, in this issue), RLS (Raman spectrometer; Rull *et al.,* 2017, in this issue), and Mars Organic Molecule Analyser (MOMA; Goesmann *et al.,* 2017, in this issue).

PanCam will be fundamental in the day-to-day scientific operations of the rover. The images and other data products will be available to the team soon after downlink to assist with scientific decisions on where to drive next, on what targets of interest geological observations should focus on, and where and which rock units ExoMars should drill. The team intends to make images available via ESA in near real time for outreach purposes as appropriate. The central role of PanCam in these activities has been shown to be vital in many field trials already (*e.g.,* Schmitz *et al.,* 2009; Steele *et al.,* 2010; Gunes-Lasnet *et al.,* 2014).

In this paper, after presenting the team organization, we discuss the PanCam scientific objectives and instrument design, and the measurement scenario, calibration, and 3-D vision aspects.

### 1.1. Team organization

**FIG. 1.**
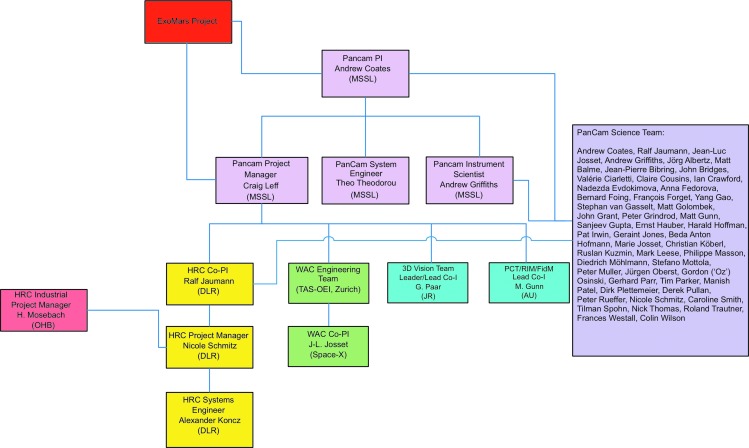
PanCam team organization.

The PanCam team organization is illustrated in Fig. 1. Mullard Space Science Laboratory (MSSL) is the principal investigator (PI) institute with overall responsibility for the instrument, supported by co-PI involvements for major hardware contributions at DLR, Germany (supported by industry, OHB), and Space-X, Switzerland (supported by industry, RUAG). In addition, there are lead-co-I roles in Austria (Joanneum Research—3-D vision team lead) and Wales (Aberystwyth University—Small Items lead). The hardware team is led by a project manager. The international science team is co-chaired by the PI and co-PIs.

In addition to the science team, which has association with each of the key components, co-I “swaps” have been initiated with the ISEM, WISDOM, and CLUPI teams, emphasizing and enhancing the collaborative nature of the mission.

## 2. Scientific Objectives

### 2.1. Contribution to overall rover mission science

The overall goals of the ExoMars 2020 rover (Vago *et al.,* 2017, in this issue) are to search for signs of past and present life on Mars and to characterize the water/geochemical environment as a function of depth in the shallow subsurface. The key new aspect of the mission is the retrieval and analysis of subsurface samples from 0 to 2 m below the oxidized surface of Mars. Rover operations include an integrated set of measurements at multiple scales: beginning with panoramic imaging and analysis of the geological context, progressing to finer-scale investigations of surface rock outcrops, and culminating with the collection of well-selected subsurface (or surface) samples to be studied in the rover's analytical laboratory.

The main scientific objectives of PanCam may be summarized as follows:

(1) Provide 2-D and 3-D geological and mineralogical context information through imaging, including construction of digital terrain models (DTMs) and their visualization;
(2) Support the selection of rover science and drilling sites by providing geological and mineralogical characterization of target rock outcrops;
(3) Geologically investigate and map the rover science traverse and drilling sites;
(4) Locate the landing site, the science sites, and the rover position with respect to local and global references by comparison and data fusion with data from orbiters;
(5) Support rover traverse planning with geological context of the environment;
(6) Image and characterize the acquired drill samples;
(7) Study the properties of the atmosphere and variable phenomena, including water and dust content of the atmosphere.

PanCam will determine the geological and geomorphological context for the remainder of the rover payload. A pair of Wide Angle Cameras (WACs) and a close-up High Resolution Camera (HRC) located at the top of the mast of the rover, together with geological, atmospheric, and red/green/blue filters, constitute a powerful camera system for planetary science. The WACs and HRC enable complementary imaging at different scales to obtain both wide-angle multispectral stereoscopic panoramic images and high-resolution color images. PanCam can view the lander upper surface and verify mechanism deployments and potentially the interaction of the drill and the rover wheels with the regolith. PanCam is the main ExoMars rover instrument for the remote characterization of the landing site's geological context, to provide detailed 3-D DTMs through stereo imaging and measure the surface Bidirectional Reflectance Distribution Function (BRDF).

The two WACs will each generate multispectral stereo images with 38.3° field of view (FOV) (horizontal/vertical), and the HRC will provide monoscopic “zoom” images with 4.88° FOV (horizontal/vertical; see Table 2); the combination provides morphological information on the rover surroundings. The WACs use multiple narrow-band filters to constrain the mineral composition of rocks and soils and the concentration of water vapor and the dust optical properties in the atmosphere.

The HRC can acquire high-resolution subset images of the wide-angle panoramas, as well as image mosaics, and furthermore enables high-resolution imaging of inaccessible locations, for example, on steep slopes such as crater or valley walls. It also allows observation of retrieved subsurface samples before ingestion into the Sample Preparation and Distribution System (SPDS) of the Pasteur payload. Combined with a Rover Inspection Mirror (RIM), placed at the front end of the rover body, engineering images of the underside of the rover chassis as well as views of the rover wheels for soil mechanics science and wheel wear can be acquired with the HRC. In addition, views of the underside of overhanging rock formations may also be acquired.

The wide-angle (WACs) and close-up (HRC) capability thus provides imaging at different scales, from submillimeter resolution (HRC) directly in front of the rover up to millimeter-to-meter resolution to effectively infinite distances. These properties allow the acquisition of information to support rover navigation. PanCam is the only scientific instrument on the rover that can generate detailed 3-D DTMs, slope maps, and similar products. This will be complemented by lower-resolution Navigation Camera (NavCam) data. It should be noted that NavCam has a 65° FOV with a 150 mm stereo baseline and is accommodated on the mast in front of PanCam tilted down by 18° (Silva *et al.,* 2013). After a drill site has been selected, using PanCam and other context information, the instrument can also view the drill tailings and thus provide complete geological context for the subsurface samples.

### 2.2. Specific scientific goals

Camera experiments are an indispensable component of the scientific payload of any surface planetary mission, whether it is a stationary lander or a mobile rover (*e.g.,* Bell *et al.,* 2003; Edgett *et al.,* 2012; Gunn and Cousins, 2016; Maki *et al.,* 2012). PanCam will study the geological diversity of individual local areas along the rover traverse and will be a significant contributor to the scientific output of ExoMars, particularly to characterization of surface geology and geomorphology, atmospheric sciences, and cartography/geodesy. Importantly, analysis of PanCam imagery will guide the selection of areas and rock outcrops for more detailed analysis and provide stratigraphic context for these sites. The application of stereo imaging will significantly enhance the ability of the science team to investigate terrain and geology through 3-D vision. DTMs and the corresponding texture maps in a visualization environment will enhance the quantitative analysis of the geomorphology and geology of target areas. In the following sections, we describe in detail the scientific objectives that will be addressed by PanCam.

#### 2.2.1. Geology and geomorphology

The major objective of PanCam imaging with respect to geology and geomorphology is the identification and characterization of rock and unconsolidated surficial units on the basis of their morphology, geometry, distribution, and physical (*e.g.,* cohesion) and spectral properties (the latter will be addressed in the next section on color investigations). Perhaps the single most important contribution of PanCam science to the mission will be to help reconstruct the geological and geomorphological history of the ExoMars study area and in particular to define the stratigraphic context. This is crucial to place sample analysis in a geological spatial and relative temporal framework. The search for biosignatures requires an understanding of the local and regional geological context (*e.g.,* Westall *et al.,* 2015), which will be interpreted mainly, but not exclusively, from imaging data. As geological context encompasses the multiscale character of natural systems, context information for planetary rover missions will not only come from *in situ* data obtained by rover instruments but also from remote sensing data acquired by orbiter instruments. Therefore, it will be essential to link observations at multiple scales by combining rover and orbiter data (*e.g.,* Golombek *et al.,* 2005, 2008; Arvidson *et al.,* 2015; Stack *et al.,* 2016). An immediate application of such combined data analysis will be the identification of landforms visible in both PanCam and orbiter images, which is key for a precise determination of the landing site location in a global geodetic reference frame (Oberst *et al.,* 1999a, 1999b; Haase *et al.,* 2012). Ideally, such combined analysis will benefit from simultaneous and coordinated observations between PanCam and orbiter instruments enabling cross-calibration for, for example, spectro-photometric investigations (ground truth; *e.g.,* Lichtenberg *et al.,* 2007; Hoekzema *et al.,* 2011; Fernando *et al.,* 2015). Importantly, the need for context information applies to all dimensions and requires 3-D products from PanCam's stereo images (*e.g.,* DTMs) as a spatial reference for other data (*e.g.,* WISDOM, ISEM; Paar *et al.,* 2015).

The search for biosignatures on Mars is considered to be most promising at sites of ancient geological strata that exhibit morphological, geological, and mineralogical evidence for sustained aqueous activity (*e.g.,* Farmer and Des Marais, 1999; Grotzinger *et al.* 2014, 2015; Vago *et al.,* 2015, 2017). The final three candidate landing sites for the ExoMars rover mission (Oxia Planum, Aram Dorsum, Mawrth Vallis) are all characterized by layered deposits suggestive of aqueous activity (depositional and/or alteration) (Bridges *et al.,* 2016a). Spatially resolved PanCam images and 3-D data products will be essential to study their sedimentary and compositional characteristics. Imaging is necessary to establish the local and regional stratigraphy (*e.g.,* Lewis *et al.,* 2008; Edgar *et al.,* 2012; Grotzinger *et al.,* 2015; Stack *et al.,* 2015), for identification of lithologies indicative of aqueous processes (such as the conglomerates in Gale Crater; Williams *et al.,* 2013), and for analyzing key sedimentological features such as cross-stratification (Grotzinger *et al.,* 2006, 2015; Lamb *et al.,* 2012) or clinoforms (Grotzinger *et al.,* 2015). High-resolution views from the HRC will allow the examination of rock textures (*e.g.,* Herkenhoff *et al.,* 2008) and the analysis of particle shape (*e.g.,* Szabó *et al.,* 2015; Yingst *et al.,* 2016) and grain size distributions (*e.g.,* Grotzinger *et al.,* 2015). Smaller grain size measurements, however, require very high image resolution and typically require a microscopic imager (*e.g.,* Jerolmack *et al.,* 2006; Edgett *et al.,* 2015), and it is likely that PanCam and CLUPI will need to work jointly toward this goal. In theory, PanCam images would also help identify potential morphological biosignatures such as microbially induced sedimentary structures (Noffke, 2009). This task, however, will require great care, as distinguishing microbially induced sedimentary structures from abiotic features is challenging (Cady *et al.,* 2003; Davies *et al.,* 2016) and needs to consider different scales of observation (Ibarra and Corsetti, 2016).

Data products derived from PanCam stereo images will permit the quantitative analysis of geological and geomorphic features of interest. A prime example is stratal layer geometry (thickness and attitude, *i.e.,* strike and dip), which can be determined from rover stereo data (*e.g.,* Metz *et al.,* 2009; Stack, 2015). At larger scales, the geometry of surfaces can be investigated by combining rover images and DTMs derived from orbiter images (*e.g.,* erosional unconformities; Watkins *et al.,* 2016). The use of 3-D data is not restricted to sedimentary rock outcrops but will support the study of all surface phenomena. For instance, it also helps to constrain eolian processes (*e.g.,* prevailing paleo-wind directions can be reconstructed from ventifact geometry; Bridges *et al.,* 2014) and quantitatively analyze volcanic landforms (Squyres *et al.,* 2007; Manga *et al.,* 2012). Rock populations have been studied at all previous landing sites (Golombek *et al.,* 2012), and PanCam images and 3-D data will be used to characterize their properties and to constrain their provenance (Craddock and Golombek, 2016).

Rover images and 3-D data can also support the study of physical properties of rocks (Nahm and Schultz, 2007; Okubo, 2007) and soils, for example, by viewing the disturbance of the soil by the mechanical interaction with a robotic arm or with rover wheels (*e.g.,* Moore *et al.,* 1987; Arvidson *et al.,* 2004). Rover telemetry data in combination with rover image data have been used by Arvidson (2015) and Arvidson *et al.* (2017) to analyze the mechanical properties of the soils traversed by the Mars Science Laboratory (MSL) rover, Curiosity.

In the case of ExoMars, PanCam will also image the growing accumulation of loose drill material around the borehole to examine its physical properties (*e.g.,* the angle of repose) and mineralogical properties. Indeed, as the MSL rover has shown, the analysis of drill powders provides important information on rock properties below the ubiquitous dust layer and on the weathering profile with depth (*e.g.,* Treiman *et al.,* 2016). Importantly, PanCam multi-angle and spectral data (see below) will also enable the study of spectro-photometric properties of the traverse area (Johnson *et al.,* 2008, 2015).

#### 2.2.2. Rock and soil composition and mineralogy

One of the great successes of the Curiosity rover has been the identification of igneous rocks present as clasts from conglomerates of the fluvio-lacustrine system, for example, the Hottah Facies in Gale Crater (Williams *et al.,* 2013). For instance, ChemCam compositional analyses together with complementary Mastcam, MAHLI, and ChemCam RMI imagery have shown the presence of feldspar-rich trachybasalts (Sautter *et al.,* 2015; Bridges *et al.,* 2016b), indicating that the Gale catchment contained fractionated igneous rocks in addition to more primitive basalts. PanCam will likewise be able to reveal igneous textures and felsic/mafic mineral proportions, either within clasts in sediments or by imaging of *in situ* lava flows that have been hypothesized for parts of Oxia Planum (Quantin *et al.,* 2015).

**FIG. 2.**
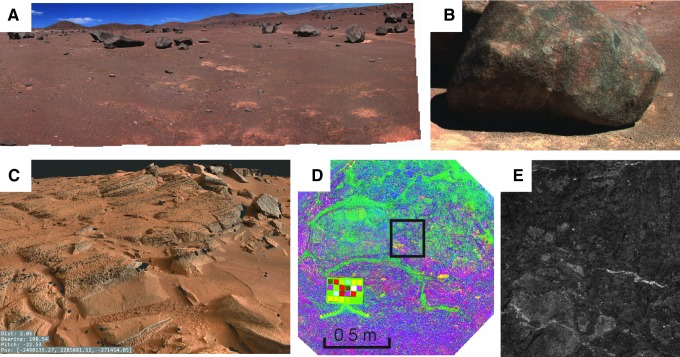
PanCam data products. (**A**–**B**) Color panorama and close-up color WAC image from the SAFER campaign in Chile (Gunes-Lasnet *et al.*, 2014). (**C**) DTM of Shaler outcrop on Mars constructed from MSL Mastcam images using the prototype 3-D vision software for PanCam. (**D**–**E**) False-color multispectral image of pillow basalt outcrop in Iceland with diagenetic mineral veins and associated HRC image (Harris *et al.,* 2015).

A first-order assessment of the composition of surface materials, including level of oxidation, hydration, and extent of chemical alteration, will be provided by PanCam WAC and HRC color images and selected WAC multispectral data. Analysis of narrowband multispectral images using the “geology” filters (Table 3) will be used to determine spectrally distinct units that can be correlated with structural features identified with WAC DTM data products (*e.g.,* Fig. 2C), building up a picture of sedimentary or volcanic structure, local stratigraphy, and geochemical evolution. This approach has been used previously to great effect using image data sets from the Mars Exploration Rover (MER) Pancams (*e.g.,* Farrand *et al.,* 2006, 2014; Rice *et al.,* 2010). Since the deployment of 12-band “geology” multispectral imaging on NASA Pathfinder (Smith *et al.,* 1997), subsequent missions have gradually modified the distribution of their geology filters across the 440–1000 nm wavelength range (Gunn and Cousins, 2016). Due to the exobiology focus of the ExoMars mission, the PanCam 12-band geology filter set was redesigned for the specific purpose of detecting minerals that had formed in the presence of liquid water (*e.g.,* clays), while maintaining the ability of PanCam to also detect Fe^2+^- and Fe^3+^-bearing mineral phases common to Mars (Cousins *et al.,* 2010, 2012), for which this wavelength range is most sensitive. While these modified filters cover different center wavelengths, many are within 3–9 nm of those flown on previous missions, including the 432, 535, 601, 673, 904, and 1009 nm filters on board the MSL Mastcam and MER Pancams (Anderson and Bell, 2013). This enables spectral observations from previous missions to be correlated with those acquired with ExoMars. Analysis of these spectral bands (Table 1), together with the other data sets produced by PanCam (and IR reflectance spectra from ISEM—see Korablev *et al.,* 2017, in this issue), will enable the identification of evidence of the paleoenvironment's habitability (see Harris *et al.,* 2015), forming a major step in deciding where to drill. The ability of PanCam multispectral data products to characterize and distinguish between different mineralogical units produced by argillic alteration of basalt was demonstrated during a field deployment of a PanCam emulator on Mars analog terrains in Iceland (Harris *et al.,* 2015).

**Table 1.** Spectral Parameters for ExoMars PanCam

*Spectral parameter*	*Abbreviation*	*Mineralogical application*
530 nm band depth	BD530	Ferric minerals, namely hematite, and degree of oxidation
Slope between 530 and 610 nm	S530-610	Ferric minerals and dust
900 nm band depth	BD900	Strength of NIR absorption, related to ferric minerals
Slope between 740 and 1000 nm	S740-1000	Strength and position of NIR absorption in ferrous minerals
Slope between 950 and 1000 nm	S950-1000	Beginning of the hydration band at ∼1100 nm
740 nm/1000 nm ratio	R740/1000	Ferrous minerals
670 nm/440 nm ratio	R670/440	Ferric minerals and dust
610 nm band depth	BD610	Can indicate goethite development
950 nm band depth	BD950	Related to hydrous minerals, some clays and silicates
Slope between 440 and 670 nm	S440-670	Related to degree of oxidation

Adapted from the works of Rice *et al.* (2010), Anderson and Bell (2013), and Harris *et al.* (2015).

Context information is essential for the successful interpretation of multispectral data, and other rover missions have used spatially highly resolved imaging data as context for multispectral spot observations. For example, the main camera on the MERs, Pancam (Bell *et al.,* 2003), provided such context for observations made by the Miniature Thermal Emission Spectrometer (Mini-TES; *e.g.,* Christensen *et al.,* 2004). More recently, the ChemCam instrument on the MSL rover Curiosity was equipped with a Remote Microscopic Imager (RMI) to provide geological context for the Laser-Induced Breakdown Spectrometer (LIBS) measurements (Maurice *et al.,* 2012; Le Mouélic *et al.,* 2015). In the same way, PanCam's HRC will not only provide context for PanCam's WAC color images but also for the ISEM spectrometer (Korablev *et al.,* 2017, in this issue). To ensure a common imaging direction and accurate positioning, the optical axes of the HRC and ISEM are coaligned.

#### 2.2.3. Landing site investigations

For the 2020 ExoMars launch, the landing site will be selected from Oxia Planum and one of either Aram Dorsum or Mawrth Vallis (Bridges *et al.,* 2016a). There are a number of hypotheses for each of the landing sites that will be investigated with PanCam and the other instruments. The final landing ellipse dimensions and azimuths have not been firmly determined for a 2021 landing, but current planning uses a semimajor axis of 50–60 km (Bridges *et al.,* 2016a).

##### 2.2.3.1. Oxia Planum (18.14°N, 335.76°E)

Oxia Planum is thought to be a layered, clay-rich Noachian terrain at the southeastern margin of the Chryse region. A long-lived aqueous system is suggested to have existed in Oxia Planum, including the distal deposits of an early Hesperian sedimentary fan that may represent an ancient deltaic sedimentary body (Quantin *et al.,* 2015). Several valley systems converge at Oxia Planum, with the proposed delta being at the outlet of Coogoon Valles (Quantin *et al.,* 2016).

In addition, Oxia Planum has an ancient, finely layered clay-bearing unit with surfaces that have been exposed as recently as 100 Ma (Quantin *et al.,* 2015). That region, in the middle and western part of the ellipse, is thought to be part of a 200 m thick phyllosilicate unit, providing primary scientific targets throughout the landing ellipse (Quantin *et al.,* 2014). These deposits have similarities with other phyllosilicate deposits, such as those at Mawrth Vallis, that occur throughout the wider western Arabia Terra and Meridiani region (Noe Dobrea *et al.,* 2010), suggesting that alteration processes could have been intense over this entire region (Poulet *et al.,* 2005).

The region also includes a 20 m thick Amazonian capping unit of uncertain, but possible volcanic, origin (Quantin *et al.,* 2014). The regions of the phyllosilicate unit that are closest to the capping unit have the strongest CRISM (Compact Reconnaissance Imaging Spectrometer for Mars) signatures for Fe/Mg phyllosilicates (Quantin *et al.,* 2014, 2015).

##### 2.2.3.2. Aram Dorsum (7.869°N, 348.8°E)

Aram Dorsum is part of an exhumed inverted fluvial channel system in a regional ancient alluvial landscape (Di Achille and Hynek, 2010; Balme *et al.,* 2015; Sefton-Nash *et al.,* 2015). There are Noachian-aged sedimentary rocks throughout the ellipse, providing scientific imaging targets for PanCam. The system has been exhumed from beneath mid/late Noachian-aged “etched terrains” (*e.g.,* Hynek and Phillips, 2008), so the region has been protected from the martian environment for what could have been several billion years.

Aram Dorsum comprises a central inverted “trunk” fluvial channel system with smaller subsidiary channels feeding into it. Traces of buried channels can be seen at various stratigraphic levels, suggestive of a long duration of sediment aggradation (Balme *et al.,* 2015; Sefton-Nash *et al.,* 2015). The main trunk channel is surrounded by what are interpreted to be floodplain sedimentary deposits (Balme *et al.,* 2014). The exposed region of the system is of the order of 10 km wide and 100 km long (Balme *et al.,* 2014). The working hypothesis for this site is that it represents a relict aggradational alluvial system that was long-lived and involved migrating fluvial channels (Balme *et al.,* 2014). Thus, it hosted a variety of alluvial depositional environments and likely comprised sedimentary rocks displaying a variety of grain sizes. The predicted long-duration nature of the system, and the potential for fine-grained alluvial sediments, makes Aram Dorsum a potentially good location for preserved biosignatures.

At the time of writing, there are very few CRISM images available for Aram Dorsum, with dust coverage obscuring the images that are available. However, at nearby Miyamoto Crater, Fe/Mg phyllosilicates were found on both sides of the channel (Marzo *et al.,* 2009; Balme *et al.,* 2015). The similarity in setting has been taken as an indication that Aram Dorsum could also contain similar phyllosilicates (Balme *et al.,* 2015)—again adding to the potential for biosignature preservation.

##### 2.2.3.3. Mawrth Vallis (22.16°N, 342.05°E)

Mawrth Vallis has abundant Fe/Mg phyllosilicates and associated layered terrains (Loizeau *et al.,* 2010, 2015). It is a well studied, mineralogically diverse site (Bishop *et al.,* 2008), which together with its stratigraphy has been taken to suggest aqueous systems and a rich aqueous history (*e.g.,* Michalski *et al.,* 2010). It is predicted that biosignatures could be retained in the phyllosilicate and hydrated silica deposits, especially as there is no evidence for mixed-layer clays that would degrade the biosignatures (Poulet *et al.,* 2014). In addition, the high clay content in Mawrth Vallis might allow organic preservation in paleosol paleoenvironments (Poulet *et al.,* 2015). Reduced paleosols are thought to be excellent scientific targets, as reducing soils cause immediate preservation and therefore can concentrate organics (Poulet *et al.,* 2014; Gross *et al.,* 2016). There are clay transitions within the landing ellipse, transitioning from Fe-smectite to overlying Al-clays (kaolinite), as can be seen in CRISM images (Poulet *et al.,* 2015).

The landing site at Mawrth Vallis is part of a large area with widespread layered deposits and evidence for draping relationships between some of them (Poulet *et al.,* 2014). Erosional landforms superposed on the clay-rich succession are also present, such as channels and small capped buttes and mesas. Furthermore, as there are few “boulder-forming” units and the thermal inertia is low, it is assumed that the region is formed at least in part from fine-grained sedimentary rocks (Poulet *et al.,* 2014). There is also a capping unit in Mawrth Vallis, which is thought to have buried the exobiology regions of interest. Like Aram Dorsum, these have been exhumed in geologically recent times (Poulet *et al.,* 2015), which improves the biosignature preservation potential.

Given the three potential landing sites, the identification and measurement of sedimentary structures and determination of the sedimentary facies and stratigraphy will be an essential part of the ExoMars rover mission. Working hypotheses for the geological context will be developed—and here, PanCam's capabilities will be central—and used to interpret the paleoenvironments that the sedimentary rocks formed in. From here, the ExoMars science team will choose which locales to target and sample using the other instruments.

The key hypotheses and questions that PanCam will be deployed to help test and address at whichever of the landing sites is chosen are as follows:

  (I) If Oxia Planum is the site, (i) What is the nature and depositional environment of the clay-bearing units? (ii) Are the Oxia clays truly the lower members of the more varied clay sequence observed at Mawrth? Is this a major regional alteration landscape? (iii) If the rover can reach the geographically constrained fan-shaped landform, is it a delta? (iv) Is the capping unit in Oxia Planum volcanic?
 (II) (i) Is Aram Dorsum an exhumed, alluvial depositional system? (ii) If so, are fine-grained floodplain and local lacustrine deposits present? (iii) Are phyllosilicates present in the Aram Dorsum region?
(III) (i) What are the depositional or diagenetic environments for the various clay mineral–hosting strata documented at Mawrth Vallis? (ii) Does Mawrth Vallis contain evidence for paleosols? (iii) What is the significance and nature of the transition from Fe-smectite to Al-clays?

#### 2.2.4. Support of exobiology

PanCam data will support the overarching exobiological objective of ExoMars through the characterization of paleoenvironments that involved sustained liquid water at the ExoMars study site. While water alone does not ensure habitability (*e.g.,* Knoll and Grotzinger, 2006; Grotzinger *et al.* 2014; Fox-Powell *et al.,* 2016), it provides the basis for current exobiological exploration of Mars (Rummel *et al.,* 2014). Since 2012, MSL Curiosity MastCam data have revealed evidence of fluvio-lacustrine sedimentary deposits (Grotzinger *et al.,* 2014, 2015) and diagenetic veins and related mineralogy (McLennan *et al.,* 2014; Nachon *et al.,* 2014; Bridges *et al.,* 2015; Schwenzer *et al.,* 2016). Critical information for construction of the stratigraphic framework of sedimentary rocks along the rover traverse and their sedimentological and paleoenvironmental interpretation has been provided by MastCam, including images of fluvial conglomerates (Williams *et al.,* 2013), a variety of cross-stratification structures, fine-scale lamination indicative of lacustrine sedimentation, and sulfate and Mg-rich veins cutting though the sediments (Grotzinger *et al.,* 2014, 2015).

MastCam observations have also demonstrated how large-scale stratigraphic relationships between geological units along the rover traverse can be understood within the context of CRISM mineralogical data (Seelos *et al.,* 2014; Grotzinger *et al.,* 2015). The role of PanCam on ExoMars will be much the same, and its combination of wide-angle, high-resolution, 3-D, and multispectral visualization (Fig. 2) will enable a thorough evaluation of the geological context for both drill-site selection and subsequent interpretation of sample analyses. Specific to ExoMars is the necessity to identify those lithologies most likely to harbor evidence for organic biosignatures (Farmer and Des Marais, 1999; Parnell *et al.,* 2007), and the degree and type of post-depositional processes that have affected them. To this end, identification of reduced, phyllosilicate-bearing sediments will be a particular priority due to the hypothesis of preferential preservation of organic matter by clay minerals (Ehlmann *et al.,* 2008). PanCam (HRC and WAC) will be an important tool in this, but this will be augmented by the use of a relevant suite of spectrometers (*e.g.,* ISEM and the Raman spectrometer). The three potential landing sites satisfy this criterion, having either spectral evidence for abundant clays (Oxia Planum, Mawrth Vallis) or geological features that suggest environments where fine-grained sediment is likely to have accumulated (Aram Dorsum).

Determination of the subsurface stratigraphic extent of candidate sampling strata through combining PanCam DTMs of rock outcrops with WISDOM data (Ciarletti *et al.,* 2017, in this issue) will also be a key input into deciding where to drill. The three landing sites are all hypothesized to contain fine-grained sedimentary rocks. Burial of such deposits is likely to have been associated with diagenesis, for example, the formation of clay and iron oxide through the dissolution of olivine, and the creation of secondary mineral veins (Léveillé *et al.,* 2014; Bridges *et al.,* 2015). The role of post-emplacement diagenetic processes is of particular importance given the ability of diagenetic processes either to mineralize (preserve) or destroy/displace organic biosignatures, in addition to UV and oxidative degradation of organic material (ten Kate, 2010). A key finding from the MSL Curiosity mission has been the prevalence of diagenetic hydrated calcium sulfate fracture-fill veins, which formed from circumneutral fluids at relatively low temperatures (<50°C) and pressures (Nachon *et al.,* 2014; Schwenzer *et al.,* 2016). Remote detection of deposits such as these with PanCam will be achieved through a combination of WAC multispectral (color, hydration band identification) and HRC (mineral vein morphology and crystal habits) data. Likewise, combined HRC and WAC multispectral data will also be used to detect other low-temperature secondary mineralization features that have the potential to trap organic matter during precipitation, including evaporitic salt phases, zeolite mineralization, and carbonates, which can then be corroborated with ISEM spectral data to identify and spatially extrapolate their mineralogy across a PanCam scene.

#### 2.2.5. Atmospheric science

The major changes on the martian surface that can be detected by PanCam are caused by eolian processes and condensation of volatiles, which directly reflect variations in the prevailing near-surface wind regime, and the diurnal and seasonal volatile and dust cycles. Atmospheric studies will concentrate on the detection of clouds, measurements of the aerosol contents, and the water vapor absorption at 936 nm (Titov *et al.,* 1999). Although currently present at only ∼30 ppm near the surface, the atmospheric water vapor distribution is vital to understanding the water exchange with the regolith and its loss to space. It may also be affected at high altitudes by any nearby crustal magnetic fields. The abundance of water vapor in the instrument line of sight will be inferred by measuring the 936 nm absorption feature, utilizing the 925 nm (continuum) and 935 nm solar filters (Table 3). By direct imaging of the setting Sun, this feature will be measured as a function of zenith angle, to probe vertical distribution in the near-surface layers. These properties of abundance and vertical profile will be retrieved with the radiative transfer and retrieval tool NEMESIS (Non-linear optimal Estimator for MultivariatE Spectral analysIS) (Irwin *et al.,* 2008). NEMESIS is a versatile tool designed for application to any planet and has retrieved vertical water vapor distributions at Mars (Lolachi *et al.*, 2007) as well as atmospheric properties of Titan (Teanby *et al.*, 2007), Saturn (Fletcher *et al.*, 2007), Jupiter (Irwin *et al.*, 2004), and Venus (De Kok *et al.*, 2011), for a range of viewing geometries.

Scattering by dust particles (aerosols) controls the quantity of light in the sky and its spectral distribution (Thomas *et al.,* 1999). Dust particles stripped from dust layers on the ground and ice, nucleated together, are important components of the aerosol load in the atmosphere. The vertical optical depth of aerosols in the atmosphere of Mars varies strongly, depending on meteorological conditions such as temperature and atmospheric pressure. The results obtained at the MER and MSL landing sites concerning atmospheric conditions were discussed by Smith *et al.* (2006), Moores *et al.* (2013), and Webster *et al.* (2013). PanCam will use images of the sky observed near sunset to determine scattering properties of the dust particles via simulation with NEMESIS (Kleinböhl *et al.*, 2011), in conjunction with observations from meteorology packages on the ExoMars landing platform. The coordination of multiple instrument observations of optical depth has been successful in constraining aerosol properties over previous missions (Lemmon *et al.,* 2004; Wolff *et al.,* 2006). The variation of scattering properties with altitude may be inferred by observing the sky after sunset, when increasingly higher levels in the atmosphere are illuminated by sunlight (Markiewicz *et al.,* 1999).

In addition to studies of dust particles and other aerosols, lander- and rover-based cameras have been used successfully to study and monitor cloud activity on Mars (*e.g.,* Moores *et al.,* 2010, 2015), and PanCam will continue this endeavor, including using automated techniques (*e.g.,* Francis *et al.,* 2014).

The observations of these atmospheric properties across the mission duration will provide temporal data, contributing to the optical depth record accumulated and reviewed by Lemmon *et al.* (2015). The collected observations made by the ExoMars rover, landing platform instruments, and Trace Gas Orbiter (TGO) will provide further insights into the characteristics and evolution of the martian atmosphere.

### 2.3. Operational planning

The planning of all rover operations relies heavily on visual information provided by various camera systems (*e.g.,* Bell *et al.,* 2003). The ExoMars rover will have a nominal lifetime of 218 sols (approximately 7 Earth months). During this period, a total drive distance of several kilometers is expected (Vago *et al.,* 2017, in this issue). To plan drives and analytical campaigns, a daily process of localization and navigation planning will be necessary. This will make use of the High Resolution Imaging Science Experiment (HiRISE) on Mars Reconnaissance Orbiter in particular, and to some extent imagery from the High Resolution Stereo Camera (HRSC) on Mars Express and the Colour and Stereo Surface Imaging System (CaSSIS) on the ExoMars 2016 TGO, together with the ExoMars rover navigation and localization cameras. Complementary to the onboard guidance navigation and control (GNC, Winter *et al.,* 2015), which performs relative localization in reaction to a predefined local path, absolute localization [both for strategic (daily) and tactical (longer-term) planning] will be performed in the Rover Operations and Control Center (ROCC) at the ALTEC premises in Turin. The PanCam 3-D vision workflow PRoViP (Paar *et al.,* 2009; see also Section 5.3 in this paper) will be able to interpret PanCam, NavCam, and Localization Camera (LocCam) data and generate 3-D vision products (panoramas and DTMs). These data are expected to be available on a daily basis to be used for scientific planning for that sol.

For absolute localization, given that high-resolution satellite DTMs/ortho images in the range of 0.5 m resolution or better are available, rover DTMs are projected into an orthorectified mosaic, which uses the XYZ location of each pixel to create a true overhead view. These mosaics are then compared with orbital (*e.g.,* HiRISE) views of the terrain to pinpoint exactly where the rover is in terms of latitude and longitude on the martian surface (Tao *et al.,* 2016). A Path Planning module based on the local DTM will facilitate the long-term route planning. A traverse-monitoring map will show areas within which the rover is allowed to enter or not.

PanCam single images, mosaics, and stereo products will provide essential higher-resolution views of potential scientific targets, sometimes with geology filters. It is envisaged that some PanCam imagery and stereo DTMs will be available in the daily cycle of downloads, with the number of PanCam products varying according to scientific priorities and data downlink rates. A key strength of ExoMars science is expected to be the rapid integration of PanCam stereo imagery with additional 3-D information into the science and drive planning process.

### 2.4. Synergies with other instruments

An important scientific property of the Pasteur payload instruments is their ability to contribute to the successive chain of observations aiming to collect the best possible samples for analysis. PanCam is the first link in this chain.

The PanCam WACs will obtain panoramic scale information needed to characterize the rover's surroundings. Without the need to move the rover, PanCam HRC, in combination with the IR spectrometer ISEM, can secure detailed, high-resolution visual and spectral data on faraway candidate targets. Armed with these results, the science team can make an informed decision regarding which geological targets to inspect at close range.

Once the rover has reached a scientifically interesting objective, for example, a sedimentary rock outcrop that could have been deposited in an aqueous environment, PanCam WAC, PanCam HRC, and ISEM can work in a concerted manner to study the target. This characterization can be used to determine which portion of the outcrop to investigate at very high resolution with CLUPI, the close-up imager accommodated on the ExoMars drill.

PanCam can also support rover operations when performing subsurface scanning maneuvers. These are predefined trajectories executed by the rover in order to construct 2-D and 3-D models using the WISDOM Ground Penetrating Radar (GPR) and the ADRON neutron detector. They allow the determination of, respectively, the subsurface stratigraphy under the rover (including the existence of potential obstacles) and the subsoil's level of hydration—both important for deciding where to drill.

PanCam and CLUPI can work in tandem to visually examine outcrops and rocks at progressively higher resolution, that is, overall views with the WACs, imaging at millimeter scale with the HRC, and detailed textural studies at tens-of-microns scale with CLUPI.

PanCam and CLUPI can also monitor drilling operations. As the drill penetrates into the ground, a mound of fines will be collected at its base. CLUPI can obtain very detailed images of these fines at 39 and 13 (drill lowered) μm/pixel (Josset *et al.,* 2017, in this issue). However, via the RIM, or once the rover has backed away, it is the PanCam HRC that can inspect the entire collection of fines, revealing any heterogeneity in the grains. This information can be compared with the borehole wall data obtained by the Ma_MISS IR spectrometer integrated in the drill tip.

Finally, the PanCam HRC and CLUPI are tasked with imaging the sample deposited by the drill on the Core Sample Transport Mechanism (CSTM)—a small drawer emerging through a port on the front of the rover to receive the sample. Once again, CLUPI can obtain high-resolution images (∼20 μm/pixel) of portions of the sample, but the PanCam HRC will achieve a top view of the complete sample prior to its delivery to the analytical laboratory for further processing and analysis.

In summary, the use of PanCam in synergy with all the rover's external instruments constitutes a major requirement for achieving the mission's scientific objectives.

Although not strictly an instrument, the rover locomotion system itself (Poulakis *et al.,* 2015) will provide PanCam with numerous scientific opportunities, for example, wheel-soil interaction studies based on mechanical properties of the terrain material that the rover will traverse, which can be assessed with PanCam and CLUPI. The results of these investigations will help improve predictions for tractive performance of flexible wheels on different terrains and slopes (the rover includes wheel slip sensors and is able to assess effective traverse progress optically). The wheels of the rover can also be used to conduct trenching observations (planned or otherwise) where PanCam and CLUPI can directly inspect (shallow) excavated material deemed interesting.

## 3. Instrument Design

### 3.1. Instrument overview

The PanCam design (total mass 2.13 kg with margin) includes the following major items:

(a) **Wide Angle Camera (WAC) pair**, for multispectral stereoscopic panoramic imaging, using a miniaturized filter wheel. The WAC units themselves are provided by RUAG and Space-X, Switzerland, and the filter wheels and drives are produced by Mullard Space Science Laboratory (MSSL), University College London (UCL).
(b) **High Resolution Camera (HRC)** for high-resolution color images. The HRC hardware is produced by OHB, Oberfaffenhofen, and DLR Institute for Planetary Research, Berlin, Germany.
(c) **PanCam Interface Unit and DC-DC converter (PIU and DCDC)** to provide a single electronic interface. The PIU and DCDC are provided by MSSL-UCL.
(d) **PanCam Optical Bench (OB) to house PanCam** and provide planetary and dust protection. The OB is provided by MSSL-UCL.

**FIG. 3.**
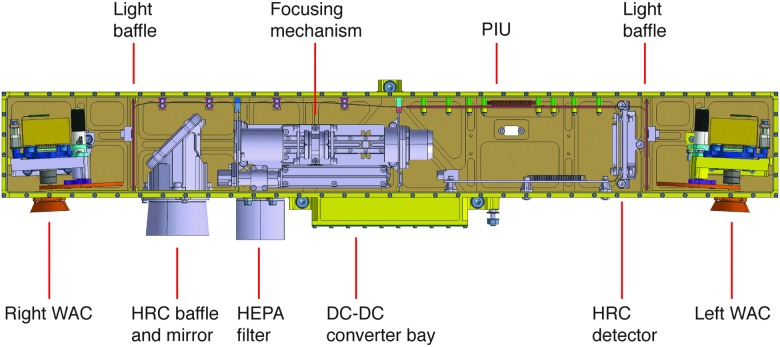
PanCam layout (MSSL).

The PanCam mechanical design is illustrated in Fig. 3. The OB is located on a rover-supplied pan-tilt mechanism at the top of the rover mast, at a height of ∼2 m above the surface (Silva *et al.,* 2013).

A summary of the main characteristics of PanCam is shown in Table 2.

**Table 2.** Main PanCam Characteristics and Resources

	*WACs ( × 2)*	*HRC*
FOV (°)	38.3 × 38.3	4.88 × 4.88
Pixels	1024 × 1024	1024 × 1024
Filter type	Multispectral Filter Wheel	On Chip RGB
Filter number	11 ( × 2 eyes)	3
IFOV (μrad/pixel)	653	83
Pixel scale (2 m)	1.31 mm	0.17
Focus	Fixed (1.0 m to ∞)	Mechanical autofocus (0.98 m to ∞)
Mass	2.13 kg (including margin)	
Power	3.4–9.2 W (including margin), depending on operating mode	

**FIG. 4.**
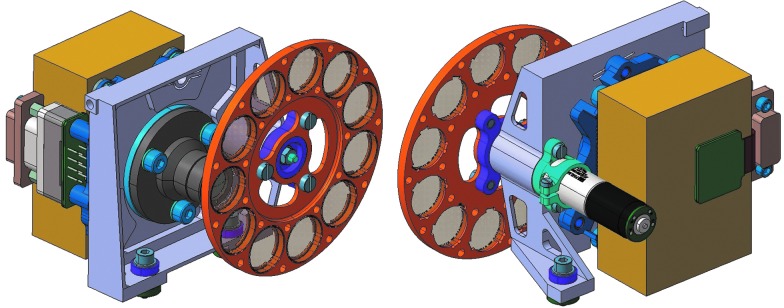
Front and rear CAD views of the right WAC assembly, showing the filter wheel and stepper motor. The left WAC and right WAC filter sets are not identical; they include RGB, narrow-band geology, and solar.

Each of the WACs includes 11 filters comprising R, G, and B color bands, a geological filter set [optimized for use on Mars by Cousins *et al.* (2010, 2012)], and atmospheric filters to analyze the water and dust content in the martian atmosphere. The filter wheel and WAC system are illustrated in Fig. 4.

**FIG. 5.**
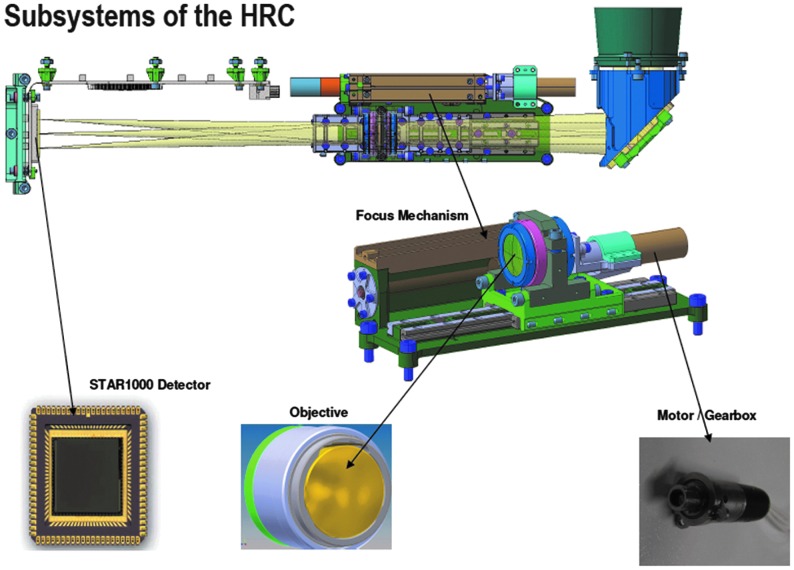
HRC subsystems: (top) schematic view and (center and bottom) elements (DLR/OHB).

The HRC includes R, G, and B filters bonded to the detector chips to provide color information. The optical path is housed within the OB structure and comprises a baffle and mirror arrangement, a focus mechanism, and a detector with associated readout electronics (see Fig. 5).

**FIG. 6.**
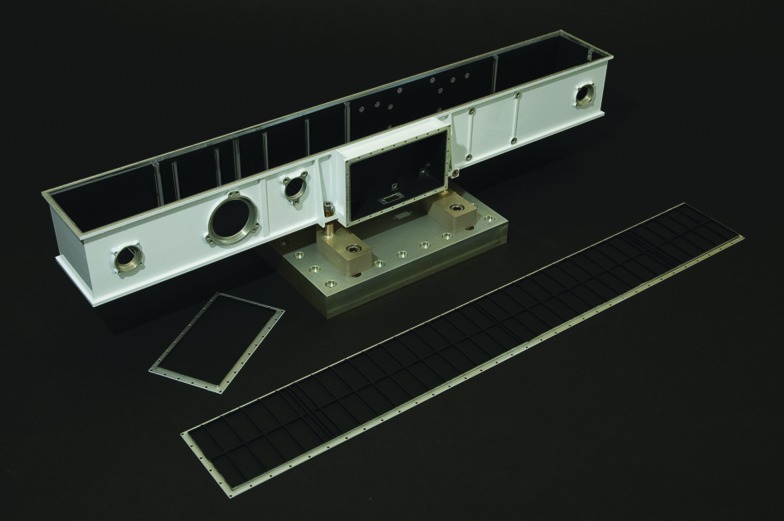
PanCam OB (STM).

The PIU is the main interface between the ExoMars rover and the PanCam subsystems, and uses an FPGA implementation. The final system component is the OB, which provides a planetary protection barrier to the external environment (including HEPA filters), as well as mechanical positioning of the PanCam components. A view of the structural-thermal model (STM) OB is shown in Fig. 6.

In addition to the four major PanCam OB-mounted components outlined above, three additional “Small Items” hardware components are part of the PanCam design to improve the scientific return and provide useful engineering data. These are the calibration target (shared with ISEM), fiducial markers, and a rover inspection mirror (see Section 3.5). We note that data formatting and compression are performed within the rover electronics system.

### 3.2. Wide angle cameras (WACs) and filter wheel

**FIG. 7.**
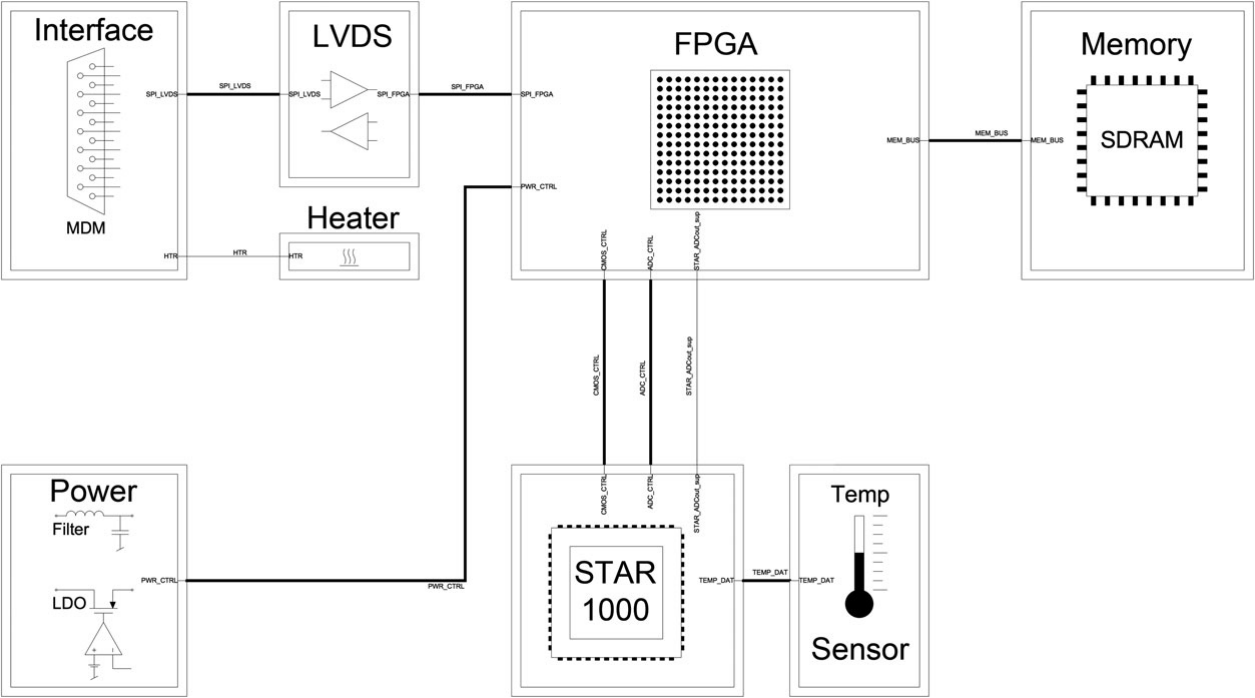
WAC block diagram showing the STAR1000 CMOS sensor with temperature sensor, the FPGA and image memory, connector, and LVDS drivers.

Each WAC contains a STAR1000 APS-based camera with wide-angle optics and a filter wheel containing 11 filters (Figs. 4 and 7).

The 1 megapixel (1024 × 1024) STAR1000 detector is digitized to 10 bits with the onboard ADC. This CMOS active pixel sensor (APS) detector was selected to reduce the number of voltage lines required and the complexity of the support circuitry (compared to a traditional CCD sensor), thus allowing lighter, more compact camera heads to be designed. The STAR1000 was developed to be extremely radiation resistant (∼100 Krad, Cos *et al.,* 2006) for long-duration geostationary missions and has flown previously as a star tracker detector (Schmidt *et al.,* 2015). It was selected at a phase in the ExoMars mission evolution when a 1.5–2 year interplanetary cruise phase near solar maximum was foreseen. The sensor is currently in flight on the Mascot lander aboard the Hyabusa 2 spacecraft (Jaumann *et al.,* 2016) and will be further qualified, for the demanding ∼200 thermal cycles it will see on the surface of Mars, as part of a life test model as part of the PanCam Assembly, Integration, and Test (AIT) phase. The product of quantum efficiency and fill factor of the STAR1000 is above 20% for most of the visible (*i.e.,* 470–710 nm) but falls monotonically to 7% at 400 nm and only 3% at 1000 nm. To compensate for these quantum efficiency limitations, the WAC filter band passes systematically increase at both long and short wavelengths (Table 3), maintaining relatively constant integration times by increasing the light energy reaching the sensor [as described in the filter selection paper by Cousins *et al.* (2012)].

**Table 3.** WAC Filter Properties for the Left Filter Wheel (Top Half) and the Right Filter Wheel (Bottom Half)

*Filter wheel position #*	*Center wavelength (nm)*	*FWHM band pass (nm)*	*Average center wavelength transmission %*	*Filter ID*
L01	570	12	98.9%	G04
L02	530	15	95.7%	**G03**
L03	610	10	95.6%	**G05**
L04	500	20	96.6%	**G02**
L05	670	12	96.2%	**G06**
L06	440	25	98.7%	**G01**
L07	640	100	99.3%	C01L
L08	540	80	98.8%	C02L
L09	440	120	98.3%	C03L
L10	925	5	0.0000552%	S01
L11	935	5	0.0000854%	S02
R01	840	25	98.9%	G09
R02	780	20	98.1%	G08
R03	740	15	98.3%	**G07**
R04	900	30	98.3%	**G10**
R05	950	50	99.4%	**G11**
R06	1000	50	99.6%	**G12**
R07	640	100	99.3%	C01R
R08	540	80	98.8%	C02R
R09	440	120	98.3%	C03R
R10	450	5	0.0001356%	S03
R11	670	5	0.0000922%	S04

Filter ID codes are as follows: G01–G12 = geology filters (10–50 nm bandwidth); C01L–C03L and C01R–C03R = red, green, and blue color imaging filters (left and right wheel, respectively, 80–120 nm bandwidth); and S01–S04 = solar filters (5 nm bandwidth).

The fixed-focus, *f* = 21.85 mm optics provide in-focus images from approximately 0.85 m to infinity (optimized for 1.9 m). PSF modeling indicates >80% of a point source's energy is contained within the central 15-micron square pixel. This figure is a worst-case value for the edge of the field with performance improving toward the optical axis. The data from the detector are digitized to 10 bits. Stereo images are acquired with a 500 mm baseline and 2.8° toe-in (per “eye”), optimized for stereo vision at 5 m from the rover. The broadband red color filters in both WACs are used for stereo imaging, while in the same sequence images are taken through the green and blue broadband filters from one WAC (known as “RRGB”) to produce an RGB color texture to “drape” over the 3-D terrain model recovered from the stereo data. PanCam is mounted on top of the rover deployable mast array (DMA) some 2 m above the martian surface. From this position it can be panned (around the vertical axis) ±180° and tilted (around the horizontal axis) ±90°, from the straight-ahead position. This allows the WACs to image the PanCam Calibration Target (PCT) and Fiducial Markers (FidMs) on the rover deck and science targets on the (unobscured) martian surface and in the sky above the rover.

Each WAC is composed of a gold-colored cube containing the power, memory, and sensor PCBs embedded in a protective epoxy attached to the 53° (diagonal) FOV lens. Unlike the HRC, the WAC design [incorporating over 20 years of development heritage—*e.g.,* Beauvivre *et al.* (1999), Josset *et al.* (2006), Griffiths *et al.* (2005)] calls for standalone modules containing 512 Mbits of memory storage for up to 50 images. The WAC is mounted to the OB just behind an 11-position filter wheel, turned by a stepper motor via a 64:1 gearbox (0.28° of filter wheel rotation per motor step). A Hall effect sensor detects magnets located along the periphery of the wheel (and also at the home position) to allow the PIU to determine the current filter location.

The 22 (total) filters are divided into three functional groups: 2 × R, G & B broadband color imaging filters (6), narrow-band geology filters in the 400–1000 nm wavelength range (12), and ultra-narrow-band solar filters (4) for dust optical density and water vapor absorption studies. The solar filters include a factor of ∼10^−5^ band pass attenuation to allow solar imaging without the detector saturating at even the minimum integration time. The filter center wavelength, pass band, and other properties are shown in Table 3. Since data bandwidth limits will rarely allow the use of all 12 geology filters on extended targets, ratios of certain filters (shown with bold IDs in Table 3)—for example, G03 and G05; G02, G06, and G01; and G07, G10, G11, and G12—will be used to identify diagnostic slopes, knees, or bands expected from hydrated clays, salts, or other minerals of exobiological interest (Harris *et al.,* 2015).

### 3.3. High Resolution Camera (HRC)

The PanCam HRC will be one of the few landscape-viewing cameras on Mars to be equipped with active focus capability, enabling it to reveal details near or far with about 8-fold better resolution than the WACs (and comparable to the 100 mm focal length, MastCam100 on the Curiosity rover).

In particular, HRC images will allow for high-resolution views of “Regions of Interest” within WAC wide-angle panoramas, as well as high-resolution imaging of rover-inaccessible locations on, for example, crater walls or in valleys. Combined with the RIM, placed at the front end of the rover body, high-resolution engineering images of the rover underside as well as views of the rover wheels for soil mechanics science or views of the underside of overhanging rock formations can be acquired.

The HRC is physically located inside the PanCam OB. The position of the viewing port next to the right WAC has been optimized to provide a top view of retrieved subsurface samples deposited by the drill on the CSTM.

**FIG. 8.**
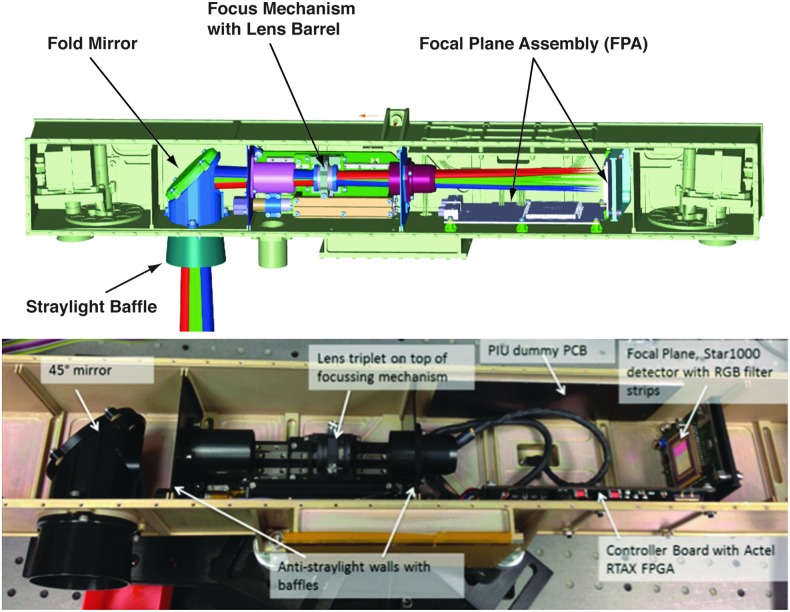
HRC CAD drawing, and Elegant Breadboard, showing HRC's components integrated into the PanCam OB.

**FIG. 9.**
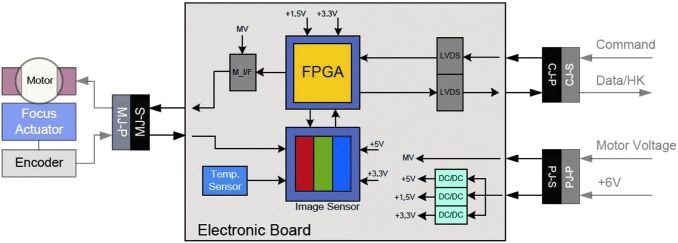
HRC block diagram showing the STAR1000 CMOS sensor with temperature sensor, the FPGA (center), connectors, power conditioning, and LVDS drivers (right) and finally the focus motor control circuitry (left).

The optical design is centered on a STAR1000 1024 × 1024 pixel CMOS detector, with 15 μm pixel size. Illuminated by a 180 mm EFL, f/16 lens (Cooke triplet), the detector enables detailed images of near and distant objects: the 4.88° square FOV takes images with a scale of ∼0.17 mm per pixel at 2 m distance and 8.5 cm per pixel at 1 km distance. PSF modeling indicates >70% of a point source's energy is contained within the central 15-micron square pixel. This figure is a worst-case value for the edge of the field with performance improving toward the optical axis. Physically, the HRC consists of the focal plane and control electronics PCBs, the focus drive stage with the lens barrel on top, the folding mirror assembly, and the entrance window (see Figs. 8 and 9). A major difference from the WAC is that the HRC lacks local image storage memory; instead, to simplify the design, the image data are transmitted directly to the PIU and buffered there. From the entrance window, the light is directed through an external and internal stray light baffle onto a 45° folding mirror. Inside the OB, an internal short wall carries a second internal baffle that directs the light onto the Cooke-triplet lens.

To enable active focusing, the lens group sits on a focus drive stage, which is mechanically actuated by a stepper motor for obtaining well-focused images between ∼1 m (distance to the sample on the CSTM) and infinity. Through a second short wall and third internal baffle, the light finally reaches the focal plane with the image sensor.

**FIG. 10.**
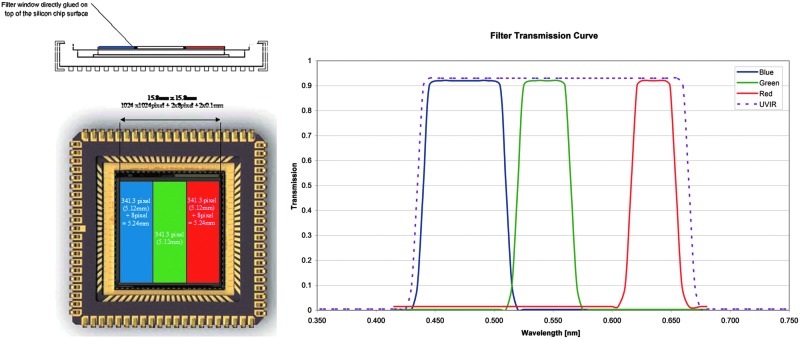
(Left) RGB strip filter, directly glued onto the active area of the STAR1000 detector. (Right) Filter transmission curve of the RGB filter strips.

The HRC uses the same monochrome APS as the two WACs. However, instead of a filter wheel, an affixed red-green-blue filter strip over the CMOS detector is used.^1^ Covering the spectral range from 440 to 654 nm (at FWHM), the three filter bands (covering, respectively, 342, 341, and 341 pixels horizontally) are blue (475 ± 35 nm), green (542.5 ± 22.5 nm), and red (635 ± 19 nm)—see Fig. 10. For color image acquisition, using the three stripe filters on the detector, the camera head has to be panned over the full FOV to mosaic each color swath across the detector FOV. We note that optical testing of the HRC elegant breadboard revealed stray light issues relating to the filter; these have been mitigated by adding a black coating to the filter edges.

Connected to the focal plane via a flex layer is the control electronics PCB, which houses all HRC electronics, including camera control, autofocus and autofocus motor control, and interface control.

To satisfy the stringent alignment requirements, the focal plane is placed on an isostatically mounted bracket. Shims between the focal plane bracket and the OB, as well as shims between the folding mirror bracket and OB, can be used to further adjust height and angular alignment during integration.

The autofocus algorithm is newly developed by the HRC team. An adapted global search strategy is used to search for the sharpest image within the focus range. The sharpness of an image is determined within a 32 × 32, 64 × 64, 128 × 128, or 256 × 256 window by Sobel filtering and calculation of the mean of the root-squared image gradients in *x* and *y* direction. The sharpness at the initial motor position is calculated by moving the motor either forward or backward depending on the current focus position, with a step length of 3 mm. Again, the image sharpness is calculated, and the motor movement direction is changed if the sharpness is lower than the initial sharpness. Then, steps of 1 mm are executed, and the image sharpness is calculated iteratively until a coarse global maximum sharpness is found. At the final stage, the step size is reduced to find the global maximum image sharpness. Although range estimation is not required for the HRC autofocus, the drive stage can be set by command at a position calculated based on range-to-target information from the WAC stereo products.

### 3.4. Optical bench (OB)

PanCam's internal components are contained in an OB. An OB was selected to provide the instrument with protection from the martian environment (*e.g.,* dust) and to provide the martian environment protection from any contaminants brought from Earth by the instrument. The OB is vented with a HEPA filter to equalize pressure but maintain cleanliness. In addition, the rigidity of the OB helps maintain a stable structure from which to acquire stereo imaging.

The OB is of aluminum construction with dimensions of 562 × 70 × 113 mm (L × H × D). The walls are machined from a single aluminum block, and afterward the interior is cut out by using electrical discharge machining wire cutting. Wire cutting allows the OB to have thinner walls than could be machined from a solid block, resulting in a lightweight rigid structure.

The base is electron-beam welded to the wall structure, and the lid is secured to the top of the bench by a series of fasteners once the internal components are mounted. In this configuration, the box makes a very stiff, extremely light monocoque structure. The mass of the OB itself, including 12% margin, is just under 780 g.

The interior of the OB is painted with a black paint (Z306) to cut down on stray light. Aeroglaze Z-306 has a reflectance of ∼8% BoL in the visible (Gilmore, 2002) and a few percent in the NIR (Ames, 1990). The exterior of the OB is coated with A276, a space-qualified white paint that aids PanCam thermal control. Equally importantly, A276 is compatible with ExoMars planetary protection guidelines; it is glossy and smooth, so it is less likely to harbor spores and other bioburden. It can also withstand dry heat microbial reduction sterilization.

### 3.5. Supporting electronics—PanCam Interface Unit (PIU) + DC-DC converter (DCDC)

The function of the PIU is to provide a single interface for all three cameras to the rover and this is achieved by using the SpaceWire RMAP interface protocol. The PIU receives the SpaceWire RMAP packets and executes them as required and forwarding the command to the cameras. Due to power constraints, only a single camera can be powered at any one time. The active camera is selected by the PIU using power switches.

Single alternating WAC operation (*e.g.,* during stereo image acquisition) is not expected to present problems maintaining operating temperature. This is because thermal modeling indicates that the low thermal conductance to the OB means that one camera is unlikely to cool significantly in the few minutes it is switched off (while the other is acquiring the matching image in the stereo pair). Alternating camera use during a sequence of stereo pairs allows heat loss from each camera to be minimized.

The PIU is responsible for control of the filter wheels. The PIU maintains locally the Rover Elapsed Time (RET) in order to allow time-stamping of HK packets and image data. In addition to time stamps, images can be identified by a unique image ID that is embedded in the image metadata and contains parameters such as the sol date and task ID. Owing to PanCam's position on the end of the mast and again to power limitations, the instrument does not have any survival heaters and follows the martian surface temperature.

Therefore, the cameras will generally be operated during the day when surface temperatures are above −40°C (to reduce power required for heating). An exception may be the solar observations just prior to sunset (sunrise observations likely being precluded by power and thermal restrictions). However, since the rover is solar powered, it is similarly limited to daytime operations when the dust opacity is low enough to enable (and any seasonal constraints allow) a suitable operating power margin to be maintained (Vago, 2012).

Given this, the PIU provides autonomous camera unit temperature control and monitoring with secondary power for the heaters. Moreover, the PIU is responsible for the control of the filter wheels. The performance and state of PanCam is monitored though the Housekeeping packets generated by the PIU. These Housekeeping packets are also used by the rover to detect any off-nominal behavior and take the appropriate actions.

A single DC-DC converter, which is housed in a dedicated compartment of the OB, provides the galvanically isolated secondary rails as required by the rest of the instrument. The DC-DC converter converts the 28 V primary power bus to three different voltages:

• 12 V, used to drive the filter wheels and the HRC focus actuator;
• 6 V, which powers the various digital components (switched to power the cameras as needed);
• 1.5 V, used by the PanCam FPGA.

The DC-DC converter incorporates a number of features such as current limiting designed to safeguard the rest of the instrument in the event of a component failure or upset.

### 3.6. Small Items

**FIG. 11.**
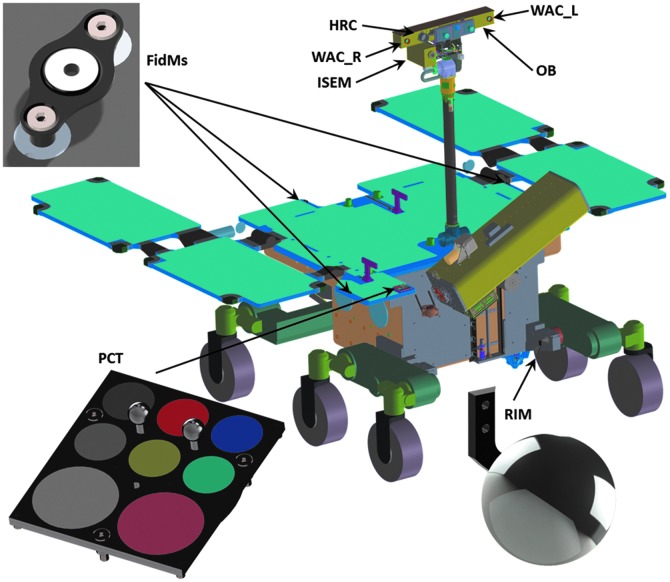
PanCam Small Items and their locations on the rover.

The PanCam “Small Items” consist of additional passive hardware to aid the surface operations of PanCam and allow *in situ* calibration. These items include the PCT, the FidMs, and the RIM. The PCT and FidMs are mounted on the rover deck and the RIM on the left-hand suspension bogie bracket as shown in Fig. 11.

#### 3.6.1. PanCam Calibration Target (PCT)

The PCT provides an *in situ* calibration target for the radiometric calibration of PanCam on the martian surface. The PCT has a mass of 36.2 g excluding mounting screws and consists of an aluminum structure with a surface area of 67 × 76 mm, which mechanically retains eight colored glass and ceramic calibration patches. Six of the calibration patches (red, yellow, green/blue, blue, and two gray) have an exposed diameter of 18 mm and will be used only for PanCam calibration. Colored glass was selected for the calibration patches, as the color has excellent resistance to UV-induced fading and damage from the moderate doses of ionizing radiation it will encounter during the mission. The surfaces of the calibration patches are processed to obtain a diffuse reflection, and the back surface is aluminized to increase the total reflectance. The glass for the six small calibration patches is standard Schott colored and neutral-density filter glass.

The white and multiband calibration patches have an exposed diameter of 30 mm and will be used for the radiometric and spectral calibration of ISEM (Korablev *et al.,* 2017, in this issue) in addition to PanCam. WCT-2065 (a rare earth doped glass developed by NIST and manufactured by Schott) is used to provide well-defined bands for spectral calibration applications in the visible and NIR. The white calibration patch is Pyroceram manufactured by the Vavilov State Optical Institute in St. Petersburg.

The calibration patches will be calibrated for absolute reflectance in total hemispherical/8° geometry and BRDF so that the angle of incident solar illumination can be compensated for. The PCT includes two shadow posts to allow the level of indirect (scattered) skylight to be assessed and aid in the determination of the illumination direction and angle.

The PCT is mounted on the front of the rover deck in a region as clear as possible from sources of shadowing and stray light and will be viewed by PanCam from an angle of ∼23° from the vertical. Measurements of the light scattered by the PCT will allow the level of incident illumination to be determined over the PanCam and ISEM spectral range. In combination with preflight calibration data, these measurements will allow PanCam images to be processed to obtain calibrated data products such as spectral parameter images and relative reflectance spectra of objects in the FOV.

Dust deposition on the PCT during the ExoMars mission will be accounted for in the data processing by developing a radiative transfer model of the PCT/dust system, building on the results of previous missions (Johnson *et al.,* 2002; Bell *et al.,* 2006; Kinch *et al.,* 2007, 2015). This will use laboratory measurements of Mars analog dust (JSC Mars-1; Allen *et al.,* 1997) on the PCT prototype as with the MER calibration target (Johnson *et al.,* 2006) and measurements of settling rates derived from the rover solar array power output (Stella *et al.,* 2005).

#### 3.6.2. Fiducial Markers (FidMs)

Together with the PCT, the three FidMs form two right-angle triangles on the rover deck to allow *in situ* geometric calibration of the PTU/PanCam system. Each FidM consists of a single piece of aluminum, anodized black and with the center section ground back to aluminum to provide high contrast. The center hole in each FidM provides a mounting point for geometric reference targets for preflight calibration activities. The FidMs have dimensions of 32 × 16 × 6.5 mm and a mass of 1.2 g excluding mounting screws.

#### 3.6.3. Rover Inspection Mirror (RIM)

The RIM is a 50 mm diameter convex spherical mirror machined and polished from aluminum, with a radius of curvature of 30 mm mounted on an aluminum bracket. The RIM assembly has a mass of 21.8 g excluding mounting screws. The RIM will be imaged by the HRC and will allow the drill spoil heap to be observed while drilling is taking place. It will also allow the underside of the rover to be observed for diagnosis in the event of problems with uneven surfaces, the drivetrain, and so on. The RIM will also allow the PanCam instrument to take “selfies” of the rover for publicity and outreach purposes.

## 4. Measurement Scenario

### 4.1. Usage of PanCam

The ExoMars mission plan is to explore the martian terrain near the landing site using a series of six experiment cycles and two vertical surveys (Vago *et al.,* 2015, 2017). The rover will target outcrops as indicators of buried rock masses that may extend below the radiation-altered and oxidized layers to depths of >1.5 m where molecular fossils may have survived for billions of years.

A “reference surface mission,” involving experiment cycles, has been designed to provide the necessary requirements for sizing the rover. The discussion in this section is based on that existing plan, which also provides a rough template for achieving the main scientific goals during the nominal surface mission. We fully appreciate that the detailed planning for Mars operations will vary significantly from this scenario. Inputs from the science and engineering teams for PanCam and other instruments will be generated on a daily basis as the mission progresses, in reaction to the martian terrain and environment and to the state of the rover.

In an experiment cycle (EC), the rover uses the Pasteur payload (over a period of 14 sols, with another 4 sols reserved for traveling between EC sites) to home in on a suitable outcrop from which to acquire samples (Vago, 2012). For particularly promising EC sites, a vertical survey can be conducted where samples are returned every 50 cm from the surface to the maximum drilling depth of 2 m.

The EC can be divided into an approach phase (3–4 sols) and a sampling phase (10–11 sols). At the beginning of the approach phase, the rover can be up to 20 m distant from a possible drill target, so a PanCam WAC panorama of up to 10 RRGB color/stereo images of the area ahead of the rover is acquired on the first sol. At the same time, HRC RGB color images and co-registered ISEM spectra (Korablev *et al.,* 2017, in this issue) of promising rocks (seen during the ∼100 m drive from the previous EC location) are acquired. From these data, an outcrop target will be chosen for the rover to approach to ∼3 m on the second sol. ADRON (Mitrofanov *et al.,* 2017, in this issue) and WISDOM (Ciarletti *et al.,* 2017, in this issue) surveys are conducted during the approach phase. A WAC image using either all 12 geology filters or selected groups (as discussed in Section 3.1) is taken of the outcrop along with a mosaic of eight HRC color images (and again eight co-registered ISEM spectra). Then on the third sol, a HRC color context image is taken of the potential drill target area to be imaged in greater detail by CLUPI. Subsequently, CLUPI will acquire up to six images (with depth from focus position information) to characterize rock microtexture and select a location to drill. Finally, on the forth sol of the approach phase, a surface sample is acquired and imaged by the HRC and CLUPI before ingestion into the ALD by the HRC and CLUPI. In this way, PanCam forms part of the “remote sensing” part of the Pasteur payload (along with WISDOM, ADRON, ISEM, and CLUPI) used to determine where to drill and acquire a sample.

During the EC sampling phase, the “remote sensing” instruments are used less frequently, with the ALD instruments taking center stage. However, assuming the surface sample results are promising, on the sixth sol a 6-position WAC RRGB mosaic is acquired to help plan a WISDOM pattern search for a location to drill deeper for unaltered samples. The WAC is used again on the seventh sol to acquire 12 RRGB images to increase the accuracy of the knowledge of rover positioning at the corners of the WISDOM pattern.

Next, on the eighth sol while drilling to at least 1.5 m depth is proceeding, the WAC is used to acquire a further 10 RRGB images for strategic planning (*e.g.,* to help determine the drive direction for the following EC). The final use of PanCam during the sampling phase is on the 10^th^ sol to image the retrieved subsurface sample with the HRC (along with CLUPI) before ingestion into the ALD.

Between the 11^th^ and 13^th^ sols, PanCam rests while the ALD instruments analyze the subsurface sample. Then, from the 14^th^ to 18^th^ sol, the rover drives to the next EC location, while conducting WISDOM and ADRON soundings. The only PanCam activity on these sols is the possible acquisition of WAC broadband red filter stereo pairs (or mosaics of stereo pairs) to provide higher-resolution views (than available with the NavCams) to help plan the rovers' course to the next EC location.

During the mission's two vertical surveys, the PanCam is mostly inactive while the rover drills at a single location. HRC images of the samples prior to ingestion can again be envisaged.

### 4.2. Instrument performance examples

The instrument performance will be determined from the integrated PanCam instrument and from calibration when the instrument has been assembled (see Section 5.1–5.2). At present, representative examples have been produced by using PanCam emulators such as the AUPE-2 Aberystwyth University PanCam Emulator 2 (*e.g.,* Pugh *et al.,* 2012; Harris *et al.,* 2015) and breadboard models, but the actual instrument performance examples are not available as yet.

**FIG. 12.**
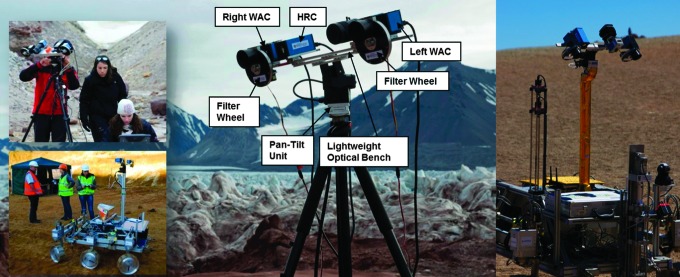
Views of AUPE PanCam simulator at tests in a Hertfordshire, UK, quarry (bottom left) and at the AMASE campaign, Svalbard.

A number of ExoMars-related field trials and tests have been performed in the last few years (see Fig. 12), including participation in recent Arctic Mars Analogue Svalbard Expeditions (AMASE) 2008–2015 (see Schmitz *et al.,* 2009; Steele *et al.,* 2010), the SAFER campaign in the Atacama Desert in 2013 (Gunes-Lasnet *et al.,* 2014), and field trials in Iceland in 2013 (Harris *et al.,* 2015). For these tests, a representative PanCam simulator was used, which was provided by Aberystwyth University. This simulator includes representative (though not the final) filter wavelengths from which spectral information may be used to study mineralogy. These campaigns have been used, in combination with teams from other ExoMars instruments, to develop working procedures representative of a mission to Mars, as well as to test instrument performance, develop calibration techniques, and pursue scientific investigations of particular areas. These included, for example, the Bockfjord Volcanic Complex and the Nordaustlandet/Palander Icecap. Scientific data from these trials are discussed elsewhere (Schmitz *et al.,* unpublished data).

Other PanCam ground tests have included “blind” geological identifications performed in the AU Mars analog facility, tests in a quarry in Hertfordshire with the Astrium UK “Bridget” prototype rover, a field trial in Shropshire in 2015, and deployment in Boulby mine (Payler *et al.,* 2017).

## 5. Calibration and 3-D Vision

### 5.1. Radiometric calibration plan

Raw PanCam images will be processed to remove image artifacts caused by the camera system and convert digital numbers into calibrated radiometric values. These processing activities will be implemented in the PanCam data processing pipeline at the ROCC. To correct camera-induced image artifacts and obtain a true representation of the scene viewed by PanCam, radiometric calibration measurements to fully characterize the camera properties will be necessary.

The radiometric calibration of the PanCam instrument will comprise a combination of component- and system-level measurements to characterize the properties of various parts of the instrument. Component-level calibrations will measure camera properties including the following: detector dark current and bias, detector linearity, detector hot/cold pixels, filter absolute transmission, PCT reflectance, and so on. System level calibrations will characterize properties of the complete system and include the following: flat-field measurements to determine the non-uniformity in the response of the optical system, measurements of system radiometric response, measurements of system spectral response and the response of the system to stray light. Dark current and flat fields will also be acquired periodically in flight to determine the effects of thermal cycling and radiation exposure on the detector response.

**FIG. 13.**
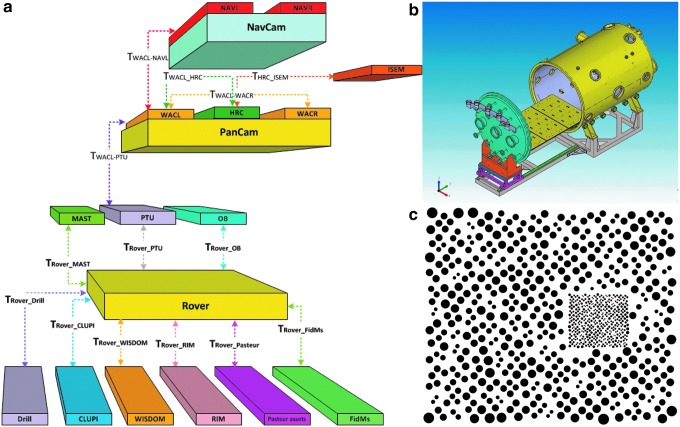
(**a**) PanCam embedded in the Geometric transformations chain of the ExoMars rover. The main element is the rover frame with various elements attached such as Drill or WISDOM (bottom). The cascade relevant for PanCam is the transform between rover and PTU, followed by the transform between PTU and WACL—depending on the pan-tilt values. WACL, HRC, and WACR are described in the PanCam coordinate system. In addition, for fusion purposes PanCam (WACL) will be cross-calibrated with NavCam and ISEM. (**b**) MSSL TVC to be used for WACs/HRC calibration in open state. (**c**) PanCam geometric calibration target design with random dot pattern for WACs (large dots) and HRC (small dots).

Many of the camera properties will be temperature-dependent, so calibration will be carried out as a function of temperature. The PanCam instrument together with calibration hardware, including an integrating sphere, will be mounted in a thermal vacuum chamber (TVC) at MSSL (see Fig. 13b) so that measurements can be carried out over the full operating temperature range of the system: −50°C to 35°C. These measurements will be interpolated to the sensor temperature recorded for the in-flight images to allow the modeled sensor response to be subtracted and estimates of remaining errors to be derived (following the methods of Bell *et al.,* 2006).

The integrating sphere will be mounted on a linear translation stage so that it can be placed in front of each of the PanCam cameras without breaking vacuum. Light sources for the integrating sphere will include white light for flat-field and linearity measurements and a tunable light source incorporating a monochromator for spectral calibrations. The light sources and a calibrated monitor spectrometer will be located outside the TVC and coupled via optical fibers. PanCam calibration measurements will be traceable to national standards laboratories (NIST) via appropriate calibration standards. These will include a calibrated light source to achieve absolute radiometric calibration and reflectance standards to determine the absolute reflectance of the PCT.

### 5.2. Geometric calibration plan

The geometric calibration of the PanCam involves all the steps required to define its components in a common coordinate system. This coordinate system will allow local reconstruction of the information collected by the PanCam components at each rover position on Mars.

To perform calibration, first each PanCam camera must be calibrated individually, second relative to each other, and then to the rest of the PanCam components. This last step is called cross-calibration.

The individual calibration of the WACs and HRC will compute the coordinates of the principal point, the focal length, and the lens distortion coefficients for each camera that is called intrinsic orientation (IO)—various representations are available. PanCam will use a derivative of the TSAI model (Tsai, 1987) with the option to convert to other well-known schemes such as CAHVOR (Di and Li, 2004). The IO of the WACs will be obtained by using one representative filter (*e.g.,* the red RGB component); distortion of other filters is measured in comparison to it by measuring the relative distortion. For HRC IO, the calibration using two different focal lengths is needed. The cross-calibration determines the geometric relationship between all PanCam components, which are mathematically expressed as 3-D-Helmert transformations, as displayed in Fig. 13a.

Changes in the IOs of the WACs and HRC will be observed by executing several temperature transitions in vacuum. Further exhaustive tests will be conducted in ambient conditions outside the TVC to assess the dependency of the filter wavelengths on the WAC IOs and the stability of the WAC and HRC IO and relative orientation after rotating the cameras around the Pan and Tilt Unit (PTU).

The original plan to bring a full setup of 3-D target points, measured by an optical coordinate metrology system (*e.g.,* Vicon), into the calibration clean room was abandoned due to planetary protection and laboratory space reasons. Instead, a flat calibration target that fulfils the planetary protection requirements, that is, appropriate for dry heat microbial reduction, will be used. It consists of an aluminum target containing a set of randomly distributed points in different scales to fit the WAC and HRC needs simultaneously, as displayed in Fig. 13c. Furthermore, in contrast to up-to-date approaches of targets with regular patterns (Bell *et al.,* 2003; Edgett *et al.,* 2015), the target can cover the full image, and the points can still be automatically detected and correctly assigned to perform a fully automatic calibration using hundreds of images.

The final AIT task requires measurements of the relative calibration between the rover and OB, PTU, FidMs, and Mast. The current plan is to accomplish this by cross-calibration with NavCam and additional adjustment of the WACs versus the PTU axes by multiple overlapping image observations under varying PTU angles and overlapping observations of the rover deck and fiducial markers under varying PTU angles.

Cross-calibration with ISEM and CLUPI will be obtained by multiple simultaneous viewing of the same target (*e.g.,* the PCT), followed by an adjustment procedure. Cross-calibration to WISDOM will be done only indirectly based on the PanCam and WISDOM individual alignment in the rover coordinate system.

Verification and possible correction of the calibration on Mars will be obtained by bundle adjustment of single stereo pairs and overlapping patches of stereo panoramas.

### 5.3. 3-D vision

Three-dimensional scene reconstruction from PanCam stereo imagery is established by the processing framework PRoViP (Planetary Robotics Vision Processing). Starting with images from the instrument available via the ESA Planetary Science Archive or NASA Planetary Data System (during the mission the data will be directly from downlink sources in the ROCC), stereo matching is performed, followed by 3-D reconstruction into DTMs in various geometries, generation of an intermediate data set (“GPC”: Generic Point Cloud), combination of the DTMs into unique consistent mosaicked products, and finally the export into products to be exploited by scientists and operations personnel.

Examples of processing products and capabilities are as follows:

(a) Digital Elevation Models in spherical, cylindrical, and Cartesian coordinate space;
(b) Ortho images;
(c) 3-D meshes;
(d) Derived thematic maps of the surroundings, describing reconstruction accuracy, occlusions, solar illumination, slopes, roughness, hazards, and so on;
(e) Fusion of rover- and orbiter-based images;
(f) Fusion between WAC and HRC 3-D vision data products (*e.g.,* overlay of WAC DTM/ortho images with HRC texture).

**FIG. 14.**
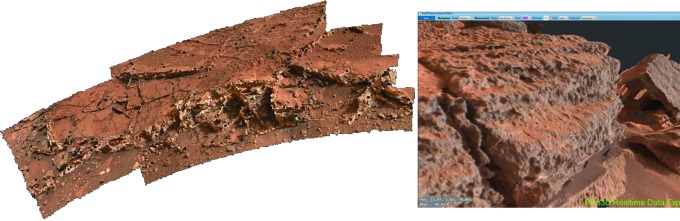
(Left) Result of MSL Mastcam processing of Garden City outcrop area taken at MSL Sol 926 and 929 (DTM, rendered by PRo3D) making use of ExoMars PanCam 3-D vision processing workflow scheme PRoViP. (Right) 3-D rendered MSL Mastcam stereo processing results at subcentimeter resolution, as appearing in PRo3D Graphical User Interface.

PRoViP has been extensively tested with various Mars mission data sets from sensors similar to PanCam, including MSL Mastcam (Barnes *et al.,* 2015a, Paar *et al.,* 2016a, 2016b) (see Fig. 14).

**FIG. 15.**
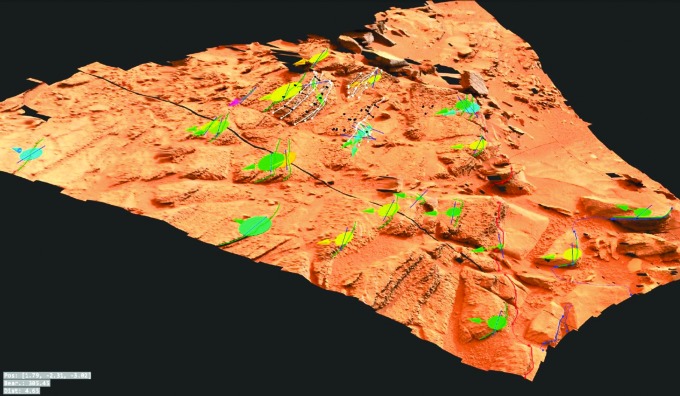
Screenshot of PRo3D showing a geological interpretation session in the Shaler area (Gale Crater, MSL mission), based on PRoViP 3-D reconstruction from a set of 99 MSL Mastcam stereo images. This detailed interpretation of the stratigraphy shows the main stratigraphic boundaries as gray lines, bedset boundaries as thick white lines, and laminations within those bedsets as thin white lines (note that the original image is in color). The dip and strike values are available directly in PRo3D in color coded by dip value and generally dip 15–20° to the southeast (validation forthcoming). The findings are consistent with those in the works of Anderson *et al.* (2015) and Edgar *et al.* (2014) in that the outcrop represents a changing fluvial environment, with recessive, fine-grained units interlayered with coarse, pebbly units. Data courtesy of NASA/JPL, image courtesy of Imperial College London, Robert Barnes/Sanjeev Gupta; www.provide-space.eu

For immersive 3-D presentation of the PanCam 3-D vision products, we will use an interactive 3-D viewing tool called “PRo3D” (Planetary Robotics 3D Viewer, Barnes *et al.,* 2015b), which allows one to virtually explore reconstructed martian terrain and perform geological analysis (Fig. 15). PRo3D builds upon the ideas of existing rendering tools used for tactical and strategic planning such as combining the rover CAD model with the 3-D reconstructed environment (Poulakis *et al.,* 2008; Cooper *et al.,* 2013; Proton, 2015) or the exploitation of image poses to generate virtual views (Howard, 2015). In addition, it goes beyond the approach of transition between panoramas and 3-D environment followed in Google Mars but gives simultaneous real-time access to different resolutions from planetary to microscopic level and therefore allows an interactive fusion of rover image products and orbiter DTMs (Paar *et al.,* 2015), closing the loop between 3-D vision processing and immersive 3-D geology. Various measurement and annotation tools are provided to

(a) Delineate geological boundaries,
(b) Obtain the true dimension of geological features,
(c) Obtain linear and projected distances between surface points,
(d) Calculate dip and strike values of stratigraphic layers.

## 6. Conclusions

We have described the scientific objectives of PanCam, its design, and how it will be used, as well as calibration methods and 3-D vision capabilities. PanCam has several powerful novel capabilities in terms of Mars camera deployment:

• The WAC spacing, 50 cm, gives excellent stereo reconstruction
• The WAC-HRC combination allows rock texture to be superimposed on the excellent DTMs
• The WAC filters have been specifically designed to reduce uncertainty in the identification of water-rich minerals
• WAC will perform atmospheric science measurements, that is, water vapor and dust determination, cloud monitoring
• The HRC will provide rock texture information and will be able to view drill tailings, samples, and underneath the rover
• PanCam will perform synergistic work with other context instruments (ISEM CLUPI, WISDOM, ADRON, and Ma_MISS)

In summary, PanCam will be a highly capable scientific camera system for the martian surface with an excellent anticipated scientific performance for geology, using filters selected for accurate identification of water-rich minerals and for atmospheric science and exobiology.

## Abbreviations Used

ADRON: Autonomous Detector of Radiation Of Neutrons
AIT: Assembly, Integration, and Test
ALD: Analytical Laboratory Drawer
AMASE: Arctic Mars Analogue Svalbard Expeditions
APS: active pixel sensor
BRDF: Bidirectional Reflectance Distribution Function
CLUPI: Close-UP Imager
CRISM: Compact Reconnaissance Imaging Spectrometer for Mars
CSTM: Core Sample Transport Mechanism
DTM: digital terrain model
EC: experiment cycle
FidMs: Fiducial Markers
FOV: field of view
HiRISE: High Resolution Imaging Science Experiment
HRC: High Resolution Camera
IO: intrinsic orientation
ISEM: Infrared Spectrometer for ExoMars
Ma_MISS: Mars Multispectral Imager for Subsurface Studies
MER: Mars Exploration Rover
MSL: Mars Science Laboratory
MSSL: Mullard Space Science Laboratory
NavCam: Navigation Camera
NEMESIS: Non-linear optimal Estimator for MultivariatE Spectral analysIS
NIR: near infrared
OB: Optical Bench
PCT: PanCam Calibration Target
PI: principal investigator
PIU: PanCam Interface Unit
PRo3D: Planetary Robotics 3D Viewer
PRoViP: Planetary Robotics Vision Processing
PTU: Pan and Tilt Unit
RIM: Rover Inspection Mirror
ROCC: Rover Operations and Control Center
STM: structural-thermal model
TGO: Trace Gas Orbiter
TVC: thermal vacuum chamber
UCL: University College London
WAC: Wide Angle Camera
WISDOM: Water Ice and Subsurface Deposit Observation On Mars

## Notes

^1^ The detector for the flight HRC will be changed to a color Bayer detector and the filter strip removed, subject to successful qualification.
